# Physical exercise and mobile phone addiction in college students: negative emotions and coping styles as chain mediators

**DOI:** 10.3389/fpsyg.2026.1933714

**Published:** 2026-10-14

**Authors:** Shuwen Guo, Wenxia Tong, Xingbo Zhou, Sen Wang, Panyi Shen, Shuqiao Meng

**Affiliations:** Department of Physical Education, Xidian University, Xi'an, China

**Keywords:** chain mediators, college students, coping styles, negative emotions, physical exercise

## Abstract

**Background:**

Considering the significant risks that widespread mobile phone addiction poses to the holistic health of college students, further research is essential. This article systematically explores correlational mechanisms linking physical exercise engagement to mobile phone addiction. Furthermore, it evaluates how Negative Emotions and Coping Styles function as mediators in this relationship.

**Methods:**

A convenience sampling approach was employed to recruit 3,283 undergraduates across the first 3 years of college. Data collection took place between April and May 2025, during which participants self-reported their engagement in physical exercise, problematic mobile phone use patterns, psychological distress (manifested as depression, anxiety, and stress symptoms), as well as their preferred coping styles. All responses were gathered through the Wenjuanxing online survey platform. For the statistical analysis, SPSS 29.0 was utilized to compute descriptive statistics and bivariate correlations. Furthermore, to evaluate the sequential mediation effect, we adopted Hayes' PROCESS macro (version 4.1, Model 6), implementing a bootstrapping approach with 5,000 iterations to generate bias-corrected confidence intervals for the indirect pathways.

**Results:**

Students showed low physical exercise (M = 18.49 ± 14.90) and variable mobile phone addiction (M = 35.40 ± 14.78). Physical exercise negatively correlated with addiction (*r* = −0.83), negative emotions (*r* = −0.39), and negative coping styles (*r* = −0.39), but positively with positive coping (*r* = 0.31). Negative emotions positively predicted addiction (*r* = 0.618). Mediation analysis showed physical exercise exhibited a correlational link with lower addiction levels (direct effect = 21.54%) through three distinct indirect correlational pathways. The path through negative emotions was dominant (72.45%), while coping-related paths accounted for 1.41%−4.6%. The independent mediating contribution of positive coping styles was slightly larger than that of negative coping styles; the dominant explanatory power across both models stemmed from the pathway of negative emotions.

**Conclusions:**

Correlational results preliminarily suggest that physical exercise may be negatively associated with college students' mobile phone addiction through correlational pathways including sequential serial paths involving negative emotions and two types of coping styles. The present correlational data could provide empirical reference for developing sports-based mental health interventions.

## Introduction

Driven by booming mobile internet development, smartphones have become indispensable tools penetrating daily communication, academic study, leisure entertainment and online consumption (Li, 2024). According to the 56th China Internet Development Report, China's mobile internet users hit 1.116 billion by June 2025, rising by 11.61 million compared with December 2024, and mobile terminals account for 99.4% of all internet access channels (Center, 2025). As the primary youth user group, college students break traditional time and space restrictions in mobile phone use, forming round-the-clock immersive usage habits (Ye et al., 2025). Long-term excessive dependence gradually evolves into mobile phone addiction, characterized by uncontrolled overuse and impaired behavioral regulation (Liu et al., 2025, 2017). Existing empirical findings reveal multiple adverse outcomes: addiction is correlated with anxiety and depressive symptoms (Zhao et al., 2025), squeezes learning time and damages academic efficiency (Zhang et al., 2024), disrupts circadian rhythm and sleep quality via nocturnal screen exposure (Deng et al., 2025), and reduces face-to-face interaction competence to induce social withdrawal (Wang et al., 2025). These intertwined negative consequences hinder college students' physical, psychological and social development, hence exploring relevant influencing pathways of mobile phone addiction carries prominent practical value. Mobile phone addiction among college students represents a pressing public-health and educational concern. Although physical exercise acts as a promising protective factor against problematic mobile phone use, few studies have systematically investigated its underlying sequential psychological mechanisms.

Existing literature indicates that higher physical activity correlates with lower mobile phone addiction severity, yet relevant psychological pathways remain underexplored. A study confirmed close interrelations between physical activity patterns and maladaptive digital engagement in youth. Although their study centered on exercise addiction rather than mobile phone addiction, their results support the view that physical activity related factors and dysfunctional digital behaviors are intertwined among young people, which resonates with our framework (Giancola et al., 2025). Drawing on Self -Determination Theory (SDT) as the core framework, insufficient satisfaction of autonomy, relatedness and competence needs is associated with negative emotions, which further foster maladaptive coping, with mobile phones functioning as a compensatory outlet elevating addiction risk. Emotion Regulation Theory and Social Conflict Theory offer only partial supplementary support for segments of this pathway. This study explores whether negative emotions and coping styles serve as sequential mediators within this association, to provide empirical references for targeted prevention and intervention among college students. Physical exercise refers to purposeful physical exercise—spanning fitness, leisure, rehabilitation, and mental training—performed to improve physical conditioning, support psychosomatic health, and sustain or enhance bodily performance (Xi, 2004). Existing scholarly work shows routine physical training is associated with lower odds of mobile phone addiction among college students (Liu et al., 2024a). Self-Determination Theory holds that unmet innate psychological needs will drive individuals to seek compensatory behaviors, which provides a core theoretical perspective for understanding mobile phone overuse. Autonomy, relatedness, and competence constitute the three innate psychological needs identified by Self-Determination Theory (Ryan and Deci, 2000, 2017). The core premise of SDT highlights that adequate satisfaction of these three basic psychological needs is critical for individuals' healthy psychological development; unsatisfied needs will prompt people to seek alternative ways for compensatory satisfaction. Various mobile applications can virtually fulfill the three needs: users gain autonomy through freely choosing online content, build interpersonal relatedness via instant social communication, and obtain a sense of competence from game tasks or online achievements. Therefore, mobile phones become an accessible compensatory outlet for college students whose core needs cannot be satisfied in real life, which raises the risk of excessive mobile phone dependence and addiction (Tong et al., 2022). Physical exercise participation among college students is associated with lower mobile phone use tendency and greater fulfillment of three fundamental psychological needs, alongside reduced addiction potential in correlational patterns. Given mobile phones' capacity for information retrieval, social interaction, and gaming as online media, mobile phone addiction mirrors internet addiction mechanisms. Research substantiates physical exercise as an effective intervention for internet addiction (Li et al., 2024b; Ye et al., 2021), Theoretical analysis implies regular physical exercise may correlate with lower mobile phone addiction tendency. Thus, this study examines Hypothesis 1: Increased physical exercise frequency correlates with less mobile phone addiction among college students.

The concept of negative emotions captures the full spectrum of aversive emotional experiences, ranging from anger and contempt to fear and sadness, all of which signify an individual's subjective sense of distress and displeasure (Lin et al., 2017). Evidence supports a robust negative correlation between physical exercise and negative emotions. Greater physical exercise is associated with better adolescent emotional health, particularly lower depressive symptoms (Wang et al., 2024); Similarly, regular physical exercise correlates with reduced negative emotions, emotional ability, and psychological distress, alongside a lower likelihood of psychological disorder onset (Liu et al., 2024b). Social Conflict Theory posits that physical exercise may help individuals release aggressive instincts, alleviate emotional blockages and reduce negative emotion generation (Xie et al., 2023). From the perspective of Self-Determination Theory, unmet psychological needs are also an important source of negative emotions. Negative emotions show strong statistical associations with mobile phone addiction (Shen et al., 2024). Neurophysiologically, Physical exercise upregulates catecholamine and endorphin release, specifically dopamine and norepinephrine. These neurochemicals modulate emotions-related brain regions in the central nervous system, leading to attenuation of negative emotions, enhancement of stress coping mechanisms, and subsequent elevation of positive emotional experiences (Gao et al., 2012). Research has revealed that negative emotions and mobile phone addiction are significantly positively interrelated, such that individuals reporting higher levels of negative emotions exhibit an elevated likelihood of developing mobile phone dependence (Chen et al., 2025). As a negative emotional experience generated when individuals face stress and adverse stimuli, negative emotions are associated with a greater tendency among college students to seek psychological solace online (Zhu et al., 2023). During the college stage, where self-control capacity is not yet fully developed, this escapist pattern correlates with higher mobile-phone-addiction tendency (Dong et al., 2024). Consistent research confirms that negative emotions positively predict mobile phone addiction severity. Among 892 college students, cross-sectional findings link elevated negative emotions to increased internet addiction risk. Specifically, severely addicted individuals reported substantially elevated anxiety and depression levels compared to non-addicted peers, and additional analysis confirmed that the addictive behaviors were primarily mobile phone-based (Chen and Zhang, 2024). Thus, we propose H2: Negative emotions mediates the association between physical exercise engagement and mobile phone addiction vulnerability.

Coping styles constitute a core indicator of individual adaptive functioning, referring to the cognitive and behavioral strategies people adopt to respond to setbacks and environmental stressors (Xie, 1998). Scholars widely categorize coping styles into two types: positive coping and negative coping. Positive coping focuses on proactive problem-solving, including tackling difficulties directly, seeking social support and conducting rational cognitive appraisal. Negative coping, by contrast, centers on avoidance-oriented behaviors, such as evasion, reality denial and escapist fantasy (Fan et al., 2025; Li et al., 2022). Rooted in Self-Determination Theory, regular physical exercise exerts profound effects on individuals' psychological functioning and stress coping mechanisms. Physical activity fulfills basic psychological needs for autonomy, competence and relatedness, accumulates positive psychological resources, and enhances individuals' perceived stress tolerance and emotional regulation competence. These psychological improvements fundamentally reshape people's coping tendencies when confronted with adversity, driving them to prefer constructive and proactive coping strategies rather than passive avoidance. A wealth of empirical evidence supports this theoretical logic: sustained physical activity is closely linked to long-term adaptive stress management patterns (Wu et al., 2020). College students who maintain regular exercise develop more mature psychological defense mechanisms compared with sedentary peers (Cheng et al., 2023). Extant research has also confirmed a robust association between coping styles and mobile phone addiction. Adaptive coping can effectively buffer the risk of problematic mobile phone use, while maladaptive coping plays the opposite role (Zu et al., 2016). Specifically, physical exercise levels correlate with individuals' stress management capacity and their reliance on maladaptive coping patterns. When people no longer resort to negative coping to relieve stress, they are less likely to depend on mobile devices to obtain instant emotional comfort or escape from real-life pressure. Such theorized mechanism may explain the observed correlational pattern regarding mobile phone addiction (Zhang et al., 2025). Meta-analytic findings further validate this correlation across college student samples (Lei et al., 2017): individuals who consistently apply positive coping to manage life stress can reduce avoidant behaviors and lower addiction risks (León Méndez et al., 2024), whereas long-term engagement in maladaptive coping such as self-blame, fantasy and evasion significantly increases vulnerability to mobile phone addiction (Chen et al., 2025). As highlighted by Self-Determination Theory, satisfaction of basic psychological needs is hypothesized to reshape individuals' coping style preferences. Synthesizing the above theoretical interpretations and empirical evidence, we propose the following hypotheses: H3: Coping styles mediate the relationship between physical exercise and mobile phone addiction. H3a: Positive coping styles mediate the association between physical exercise and mobile phone addiction among college students. H3b: Negative coping styles mediate the association between physical exercise and mobile phone addiction among college students.

College students are undergoing the transition from campus life to social engagement, and are frequently exposed to academic burdens and interpersonal conflicts, which easily trigger persistent anxiety, frustration and other negative emotions. Consistent with classic stress process theoretical logic, affective responses such as negative emotions emerge as immediate psychological reactions to stress, and these emotional states subsequently govern the adoption of coping strategies rather than the reverse. This temporal theoretical rationale underpins our model ordering. Prior studies have demonstrated that negative emotions are strong predictors of coping tendency (Meng et al., 2025): prolonged negative affective states erode individuals' motivation for active problem-solving, and push them toward avoidant coping and escapist fantasy (Gu et al., 2024). In contrast, positive coping strategies—including seeking social support, making rational study plans and cultivating hobbies—help restore psychological resilience and accelerate emotional recovery (Fan et al., 2025). It is critical to clarify a conceptual distinction here: physical exercise acts as an exogenous predictive variable in our research model, rather than a specific form of positive coping. The two constructs differ essentially: coping styles refer to response strategies adopted after stress occurs, while physical exercise is a stable daily behavioral pattern that shapes psychological states in advance and further influences subsequent coping choices. When academic performance falls short of expectations, the linkage between negative emotions and coping selection becomes more pronounced. Students who lack sufficient positive coping resources tend to seek instant gratification via excessive browsing on social media and online games to escape stress (He et al., 2020). Such temporary avoidance merely relieves negative emotions superficially without addressing fundamental problems. Reduced learning efficiency and degraded offline social competence will in turn amplify stress, forming a vicious cycle: negative emotions → negative coping → problematic mobile phone use, which steadily elevates the risk of mobile phone addiction (Tang et al., 2013).

Under the unified framework of Self-Determination Theory, we derive this variable sequence from need-frustration logic. As a long-term, stable daily behavior formed prior to stress, negative emotions and addictive behaviors, physical exercise is defined as the exogenous predictor in this model. We also acknowledge the bidirectional association between physical exercise and mobile phone addiction, which will be further discussed in the limitations section. Given that physical exercise functions as the starting independent variable throughout the entire conceptual model and sequentially affects negative emotions, coping styles and ultimately mobile phone addiction, we put forward the fourth hypothesis: H4: Negative emotions and coping styles sequentially mediate the relationship between physical exercise and mobile phone addiction among college students.

Numerous existing studies have confirmed the direct linkage between physical exercise and mobile phone addiction. However, most only test negative emotions or coping styles as separate single mediators; few build two parallel chain mediation models to compare positive and negative coping pathways and quantify their effect ratios. Rooted in the foregoing theoretical framework of Self-Determination Theory, supplemented by stress process logic, Emotion Regulation Theory and Social Conflict Theory, our serial mediation model is derived from the above reasoning and empirical findings. Each hypothesis is supported by corresponding theoretical and empirical evidence: H1 draws on SDT perspectives of need satisfaction and compensatory digital behaviors; H2 builds on emotion related links between exercise, negative affect and mobile phone addiction; H3 H3b are based on associations between physical activity, coping strategies and addiction risk; H4 is justified by stress process logic that negative emotions precede coping responses. To fill such research inadequacies constitutes the core innovation of the present study.

This study presents a serial mediation model (Figure 1) delineating how negative emotions and coping styles sequentially link physical exercise to mobile phone addiction in undergraduates. The derived conclusions are intended to underpin the development of targeted, evidence-based programs for mitigating mobile phone addiction in the college population.

**Figure 1:**
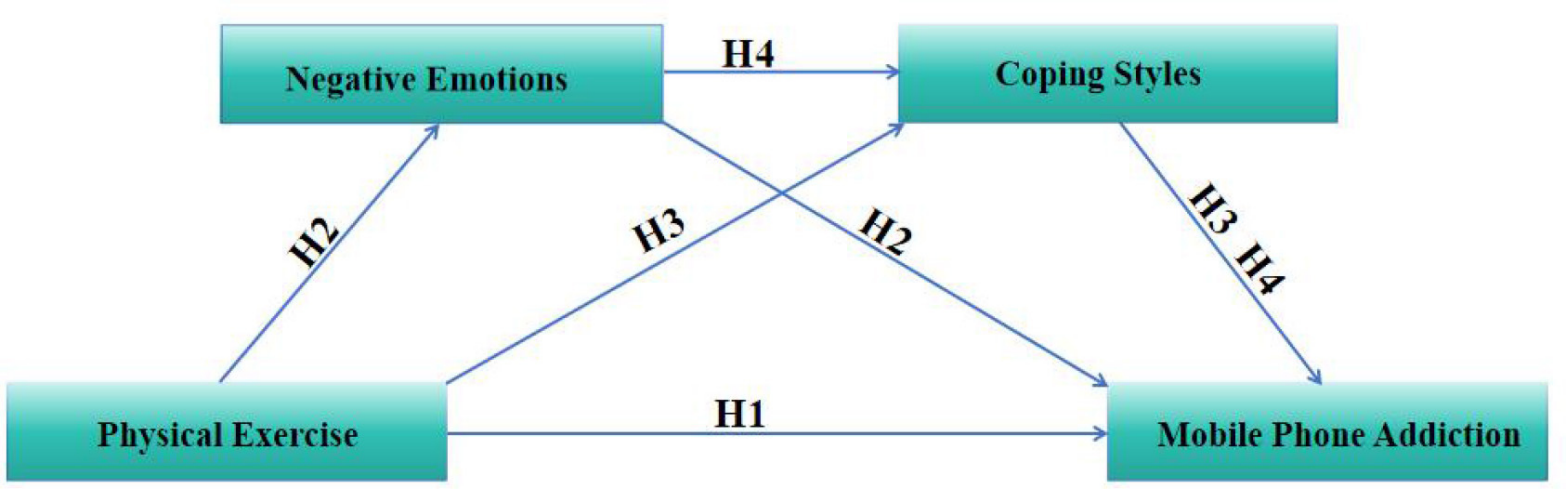
Hypothesized research model.

## Methods

### Participants

This study employed a cross-sectional design and convenience sampling method, which features easy implementation, low cost and high efficiency for data collection. The specific methodology is illustrated in Figure 2.

**Figure 2:**
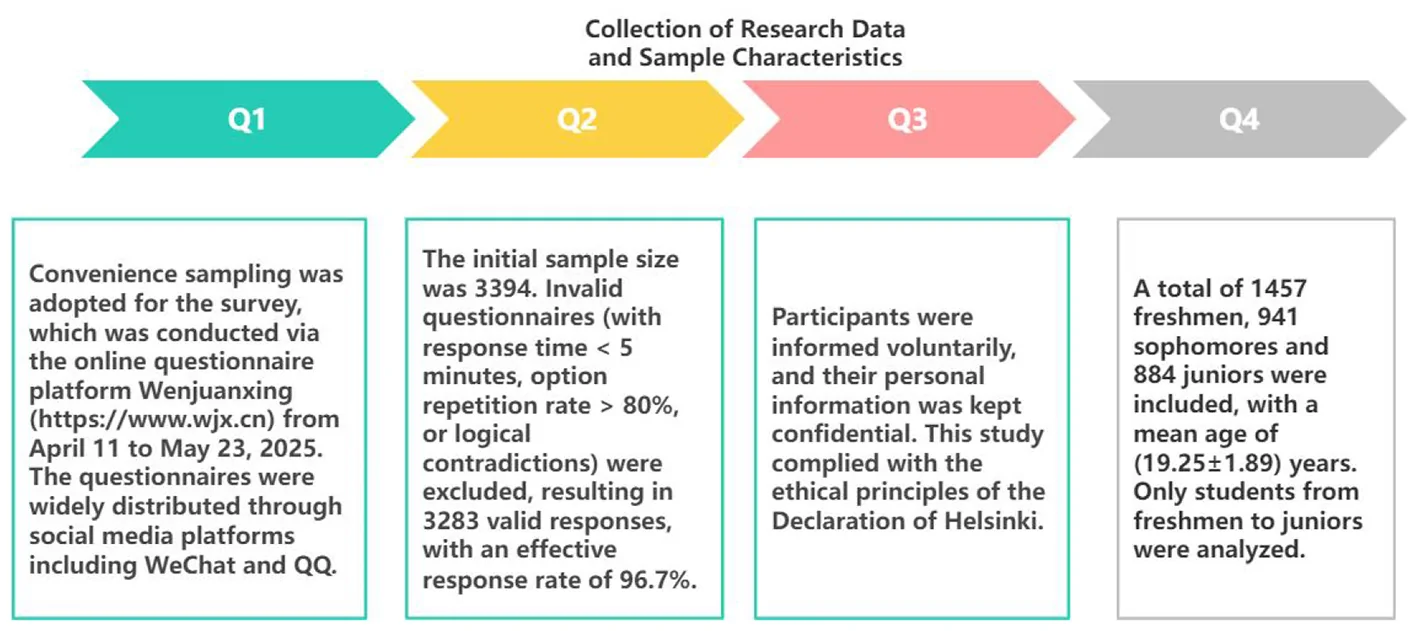
Sampling flowchart.

To verify sample size adequacy for the serial mediation model, we conducted a Monte Carlo simulation-based sensitivity analysis (Schoemann et al., 2017). Rather than relying on coefficients from our own data, we tested a range of plausible effect sizes drawn from published mediation studies in college student populations (Wang et al., 2024; Cheng et al., 2023; Ye et al., 2021). Three scenarios were specified—conservative (all paths β = 0.15), moderate (β = 0.20), and moderate-to-large (β = 0.30)—with α = 0.05 and target power ≥ 0.95 for the smallest indirect effect. Across 5,000 replications per scenario, the minimum required sample sizes were *N* ≥ 520, *N* ≥ 300, and *N* ≥ 140, respectively. Data were collected from April 11 to May 23, 2025 via the Wenjuanxing online platform, with links distributed through university and class WeChat/QQ groups; participation was voluntary and uncompensated. Implied informed consent was obtained by clicking “agree and continue” at questionnaire entry. Questionnaires completed in under 5 min, with >80% identical responses, or containing logical contradictions were excluded. Of 3,394 initial responses, 3,283 were retained (96.7% valid rate), substantially exceeding the most conservative sample requirement. The valid sample contained 1,908 male (58.1%) and 1,375 female (41.9%) undergraduates (freshmen-juniors only; age = 19.25 ± 1.89 years) across multiple disciplines. Seniors and postgraduates were excluded to avoid confounding influences. Gender and grade were controlled in subsequent analyses. No minors were recruited; all data were fully anonymized for privacy protection. This study adhered to the Declaration of Helsinki and received ethical clearance from Henan college's Biomedical Research Ethics Subcommittee (Husom2024-640).

### Measures

#### Physical activity rating scale

Physical exercise levels were measured via the Chinese-adapted Physical Activity Rating Scale (PARS-3; Liang, 1994), which gauges intensity, duration, and frequency on five-point Likert scales (1–5). Total activity scores derive from intensity × frequency × (duration – 1). Specifically, the formula is: Total Score = Intensity × (Duration – 1) × Frequency, where each term denotes the original item score. Per the original scale, this composite score inherently ranges from 0 to 100, with three predefined classification levels: Low (0–19), Moderate (20–42), and High (43–100) physical activity. Higher composite scores correspond to greater physical-exercise intensity and frequency. Scale reliability was satisfactory (Cronbach's α = 0.782). Confirmatory factor analysis indicated adequate fit: χ^2^/df = 2.856, *p* < 0.001; GFI = 0.914; RMSEA = 0.071; CFI = 0.918; NFI = 0.905; NNFI = 0.908, confirming structural validity.

#### Mobile phone addiction scale

The Mobile Phone Addiction Index Scale, revised from Leung's original Mobile Phone Dependence Scale (Leung, 2008) and further verified for reliability and validity by Hai et al. (2014), was adopted in this study to measure mobile phone addiction. The instrument comprises 17 items spanning four domains—control loss, withdrawal, escapism, and inefficiency—rated on five-point Likert scales. Mobile phone dependence was operationalized as ≥8 endorsed items plus a total score ≥40. Higher total scores denote more severe mobile phone addiction symptoms. Although this scale has been broadly employed in global studies, a comprehensive validity assessment was performed before formal application owing to the remarkable changes in college students' mobile phone usage modes. The findings indicated that the scale displayed satisfactory structural validity, discriminant validity, and internal consistency reliability in the student sample, and could effectively characterize mobile phone addiction in high-frequency usage contexts such as social media and short videos. The tool presented favorable applicability and timeliness, which provided reliable support for variable evaluation in this research. Overall reliability was strong (α = 0.91), with subscale alphas of 0.84 (control loss), 0.83 (withdrawal), 0.87 (escapism), and 0.81 (inefficiency). CFA evidenced adequate fit: χ^2^/df = 2.84, *p* < 0.001; GFI = 0.997; RMSEA = 0.07; CFI = 0.906; NFI = 0.905; NNFI = 0.908. These psychometric properties support the scale's utility for mobile phone addiction assessment.

#### Depression anxiety stress scale-21

The Depression Anxiety Stress Scales-21 (DASS-21) Simplified Chinese Version, revised by Gong et al. (2010), was used to assess negative emotions among college students. The instrument comprises 21 items spanning three subscales—depression, anxiety, and stress—seven items apiece. A four-point Likert scheme from 0 to 3 was adopted for assessment (0 stands for does not fit me at all and 3 stands for fits me very well or most of the time). Higher subscale and total scores indicate more severe symptoms of depression, anxiety, and stress. Elevated scores indicate greater depression, anxiety, and severity. In this study, Cronbach's α coefficients for the total DASS scale, depression, anxiety, and stress subscales were 0.929, 0.823, 0.823, and 0.818, respectively, indicating satisfactory internal consistency across all dimensions. Confirmatory factor analysis based on the three-factor structure yielded acceptable model fit: χ^2^/df = 2.76, *p* < 0.001, GFI = 0.906, RMSEA = 0.069, CFI = 0.904, NFI = 0.891, NNFI = 0.891. Such psychometric results support the reliable application of this scale to measure negative emotions among college students.

#### Simplified coping styles questionnaire

The Simplified Coping Style Questionnaire (SCSQ), developed by Xie (1998), was employed to assess coping strategies. This self-report tool includes 20 items and is divided into two subscales: positive coping (12 items) and negative coping (8 items). A four-point Likert system was used for evaluation: 0 = never used, 1 = used occasionally, 2 = used sometimes, 3 = used frequently. Higher scores on the positive-coping subscale reflect more frequent adoption of adaptive coping strategies, whereas higher negative-coping subscale scores indicate greater tendency toward avoidant/maladaptive coping. In this research, the scale presented satisfactory internal consistency; the Cronbach's α values were 0.948 for the positive coping subscale and 0.871 for the negative coping subscale. In addition, acceptable model fit was supported by confirmatory factor analysis: χ^2^/df = 4.25 (*p* < 0.001), GFI = 0.974, RMSEA = 0.036, CFI = 0.912, NFI = 0.908, NNFI = 0.915. These results confirm that the questionnaire possesses good reliability and validity in this study and serves as an effective tool for measuring coping styles among college students.

### Data analysis

The AVE method was applied to evaluate discriminant validity. The DASS−21 had a Cronbach's α of 0.929 and an AVE of 0.76 for negative emotions, and the negative coping subscale of the SCSQ had a Cronbach's α of 0.871 and an AVE of 0.73. Both AVE values exceeded the commonly accepted cutoff of 0.5. The squared correlation between the two constructs was 0.7225, which was smaller than their respective AVE values, confirming acceptable discriminant validity. Data analysis was conducted in SPSS 29.0. Descriptive statistics and Pearson correlation analyses were carried out on the collected data (Preacher and Hayes, 2004). To examine the mediating effects, Model 6 from the Process 4.1 macro, based on the mediation testing procedures outlined by Wen and Ye, 2014, was utilized. The mediation effect was then validated using the Bootstrap method, in which 95% confidence intervals were calculated through 5,000 repeated samplings. A statistically significant mediation effect was identified when the interval did not contain zero. The bias corrected percentile Bootstrap was used to compare specific indirect effects pairwise. Pathway differences were indicated by non-overlapping 95% confidence intervals (exclusion of zero). Common method bias was assessed using Harman's single-factor test (Podsakoff et al., 2003). Procedural measures including full participant anonymity were adopted during data collection to mitigate potential method-related bias. To avoid confounding effects of gender and grade on the research results, both variables were included as control variables in all regression analyses. This treatment effectively improved the credibility and robustness of the empirical results.

## Results

### Common method bias test

Given that all variables were measured via self-report questionnaires, we assessed potential common method bias using Harman's single factor test (Podsakoff et al., 2003). All scale items were subjected to unrotated exploratory factor analysis. The first extracted factor accounted for 35.1% of the total variance, falling below the commonly applied 40% empirical threshold, suggesting that severe common method bias was unlikely to exist. Procedurally, full anonymity was guaranteed for all participants during online data collection to reduce potential method-related bias. It should be noted that Harman's single factor test represents a relatively conservative diagnostic tool for evaluating common-method variance.

### Descriptive statistics and correlation analysis

Analyses proceeded in SPSS 29.0. Table 1 presents descriptive statistics and Pearson correlations for key variables: physical exercise, mobile phone addiction, negative emotions adaptive coping, and maladaptive coping. Assessment of distributional properties revealed that while physical exercise demonstrated positive skewness and non-normality, the other variables were approximately normally distributed. Sensitivity analysis adopting square root transformed physical exercise scores was conducted, and the direction and relative magnitude of all mediation effects remained highly consistent with original results. Benefiting from the large sample size (*N* = 3,283) and bootstrap based mediation test, our main analyses are robust to this non-normality. Pearson correlation analysis under large sample size was robust to mild non-normality, and the core mediating effect was tested by bootstrap method independent of normal distribution, which made the statistical method reasonable.

**Table 1:** Descriptive statistics and correlation analysis of variables.

Variable	M	SD	1	2	3	4	5	6	7	8	9	10	11	12	13
1. Gender	1.42	0.49	1												
2. Grade	1.82	0.83	–	1											
3. Physical exercise	18.49	14.9	–	0.47^***^	1										
4. Mobile phone addiction	35.4	14.78	–	−0.45^***^	−0.830^***^	1									
5. Loss of control	15.12	6.31	–	−0.69^***^	−0.720^***^	0.938^***^	1								
6. Withdrawal	7.82	3.85	–	0.10^***^	−0.750^***^	0.903^***^	0.766^***^	1							
7. Avoidance	6.54	3.22	–	−0.69^***^	−0.750^***^	0.861^***^	0.712^***^	0.745^***^	1						
8. Inefficiency	5.92	2.91	–	−0.17^***^	−0.810^***^	0.896^***^	0.789^***^	0.773^***^	0.733^***^	1					
9. Negative emotions	30.79	10.05	–	−0.51^***^	−0.390^***^	0.618^***^	0.561^***^	0.558^***^	0.546^***^	0.576^***^	1				
10. Depression	10.16	3.6	–	0.32^***^	−0.260^***^	0.579^***^	0.528^***^	0.519^***^	0.499^***^	0.555^***^	0.948^***^	1			
11. stress	10.55	3.7	–	0.07^***^	−0.350^***^	0.606^***^	0.548^***^	0.547^***^	0.545^***^	0.561^***^	0.958^***^	0.862^***^	1		
12. Anxious	10.08	3.28	–	−0.22^***^	−0.290^***^	0.573^***^	0.521^***^	0.521^***^	0.51^***^	0.523^***^	0.942^***^	0.834^***^	0.86^***^	1	
13. Positive coping styles	34.67	9.22	–	−0.81^***^	0.310^***^	−0.107^***^	−0.097^***^	−0.108^***^	−0.062^***^	−0.122^***^	−0.247^***^	−0.262^***^	−0.215^***^	−0.226^***^	1
14. Negative coping styles	18.83	5.44	–	0.29^***^	−0.390^***^	0.119^***^	0.098^***^	0.126^***^	0.108^***^	0.105^***^	0.850^***^	0.074^***^	0.083^***^	0.086^***^	0.525^***^

*N* = 3,283. ^***^p < 0.001. Gender was coded as a binary variable (1 = male, 2 = female), and Pearson correlations were not calculated for gender. Grade: 1 = freshmen, 2 = sophomores, 3 = juniors. Variables corresponding to column numbers: 1 = Gender, 2 = Grade, 3 = Physical Exercise, 4 = Mobile Phone Addiction, 5 = Loss of control, 6 = Withdrawal, 7 = Avoidance, 8 = Inefficiency, 9 = Negative emotions, 10 = Depression, 11 = Stress, 12 = Anxiety, 13 = Positive coping styles, 14 = Negative coping styles).

Descriptive statistical results indicated that within the sample of this study, the average score for physical exercise was 18.49 ± 14.90. Based on the grading standard of the Physical Activity Rating Scale by Liang (1994), this score represented a low exercise level, revealing inadequate physical exercise among students. The mean score of mobile phone addiction was 35.40 ± 14.78, below the 40-point threshold for dependence, suggesting that addictive symptoms were not severe overall, while the large standard deviation indicated wide individual differences. The average score of negative emotions was 30.79 ± 10.05, with subscale scores of 10.16 ± 3.60, 10.08 ± 3.28, and 10.55 ± 3.70 for depression, anxiety, and stress, respectively. The evenly distributed scores suggested a mild to moderate level of negative emotions. For coping styles, the mean scores were 34.67 ± 9.22 for positive coping and 18.83 ± 5.44 for negative coping. Following the scoring guidelines established by Xie (1998) for the Simplified Coping Style Questionnaire, the data suggest that when encountering adverse events, the college population favors positive coping styles over negative alternatives.

Pearson correlation analysis was adopted to examine the bivariate associations between all study variables. Given the large sample size (*N* = 3,283), even negligible relationships can reach statistical significance easily. For this reason, we primarily interpret results by referring to correlation coefficients (*r*) as the indicator of effect size, rather than overemphasizing *p*-values. We followed widely accepted criteria to classify correlation magnitudes: |*r*| < 0.1 indicates a weak association, 0.1 ≤ |*r*| < 0.3 a small association, 0.3 ≤ |*r*| < 0.5 a moderate association, and |*r*| ≥ 0.5 a strong association.

The correlation matrix demonstrated that all associations between core variables reached statistical significance at *p* < 0.001. In line with our pre-specified analytical strategy, we focused on the practical magnitude and direction of these correlations. Correlation patterns (Table 1) revealed: (a) Physical exercise had a strong negative correlation with mobile phone addiction (*r* = −0.830, large effect size), which was consistent with Hypothesis 1. (b) Mobile phone addiction was positively correlated with negative emotions (*r* = 0.618, large effect size) and negative coping styles (*r* = 0.119, small effect size), while it was negatively correlated with positive coping styles (*r* = −0.107, weak effect size). Negative emotions showed a strong positive correlation with mobile phone addiction (*r* = 0.618, large effect size) and a strong positive association with negative coping styles (*r* = 0.850, large effect size), and its correlation with positive coping styles was negative (*r* = −0.247, small effect size). These findings were consistent with previous empirical evidence and provided preliminary support for the serial mediation pathway proposed in Hypothesis 4 (Zu et al., 2016). (c) Physical exercise was moderately negatively correlated with negative emotions (*r* = −0.390) and negative coping styles (*r* = −0.390), and showed a small-to-moderate positive correlation with positive coping styles (*r* = 0.310).

Overall, the direction and magnitude of variable associations were in accordance with theoretical expectations and research hypotheses, which fully satisfied the fundamental prerequisites for serial mediation analysis (Preacher and Hayes, 2004). Gender and grade were controlled for in all model analyses to minimize potential confounding bias.

### Analysis of the chain mediating effect of negative emotions and coping styles

Sensitivity analyses revealed that removing both gender and grade from covariates yielded nearly identical path coefficients and effect proportions, indicating these demographic variables exerted minimal confounding impact on the overall mediation results. Although gender and grade were entered as control variables across all main regression models, sensitivity testing shows their inclusion hardly altered the core mediating pathways, so they do not act as important suppressor or confounder variables in the present study.

A serial mediation model (Table 2) examined correlational mediation pathways linking physical exercise to mobile phone addiction via negative emotions and coping styles. Mediation significance was indicated by a 95% confidence interval (CI) that excluded zero. Physical exercise was negatively associated with negative emotions (β = −0.38, *p* < 0.001) and an increase in positive coping (β = 0.211, *p* < 0.001). Negative emotions were in turn associated with lower positive coping scores (β = −0.211, *p* < 0.001) and higher addiction scores (β = 0.425, *p* < 0.001); positive coping scores were associated with lower addiction scores (β = −0.260, *p* < 0.001).

**Table 2:** Regression models for the mediating effects of negative emotions and positive coping styles.

Regression equation		*R* ^2^	Regression coefficients			*P*
Outcome variable	Predictive variable		SE	β	*t*	
Negative emotions	Physical exercise	0.144	0.012	−0.380	−31.667^***^	< 0.001
Positive coping styles	Physical exercise	0.225	0.015	0.211	14.067^***^	< 0.001
	Negative emotions	0.225	0.015	−0.211	−14.067^***^	< 0.001
Mobile phone addiction	Physical exercise	0.196	0.014	−0.260	−18.571^***^	< 0.001
	Negative emotions	0.004	0.020	0.425	21.250^***^	< 0.001
	Positive coping styles	0.005	0.0222	−0.350	−15.766^***^	< 0.001

^***^P < 0.001; This analysis has included gender as a control variable, the same applies below.

Mediation results (Table 3; Figure 3) revealed a significant total effect of −0.933 (95% CI [−1.149, −0.493]), supporting 1s inverse physical exercise-addiction relationship. The direct effect (−0.201, 95% CI [−0.462, −0.060]) accounted for 21.54% of the total, while the total indirect effect (−0.732, 95% CI [−0.902, −0.482]) explained 78.46%, confirming negative emotions and positive coping as significant mediators. Three specific pathways emerged: Path 1 (physical exercise → negative emotions → mobile phone addiction): −0.676 (95% CI [−0.882, −0.477]); Path 2 (physical exercise → positive coping styles → mobile phone addiction): −0.043 (95% CI [−0.076, −0.017]); Path 3 (physical exercise → negative emotions → positive coping styles → mobile phone addiction): −0.013 (95% CI [−0.021, −0.005]). All 95% CIs excluded zero, showing statistical significance for these indirect associations. Nevertheless, the serial-chain path (Path 3) only accounts for 1.41% of the total effect. Although this sequential path is statistically detectable in our large sample, its effect size is extremely small with limited practical explanatory power. It represents a theoretically plausible associational sequence rather than a practically effective pathway.

**Table 3:** Tests of mediation effects between physical exercise and mobile phone addiction among college students.

Path	Effect	Boot SE	Boot 95% CI	Proportion (%)
Total effect	−0.933	0.167	[−1.149, −0.493]	100
Direct effect	−0.201	0.133	[−0.462, −0.060]	21.54
Total indirect effect	−0.732	0.106	[−0.902, −0.482]	78.46
Path 1	−0.676	0.104	[−0.882, −0.477]	72.45
Path 2	−0.043	0.015	[−0.076, −0.017]	4.6
Path 3	−0.013	0.004	[−0.021, −0.005]	1.41

**Figure 3:**
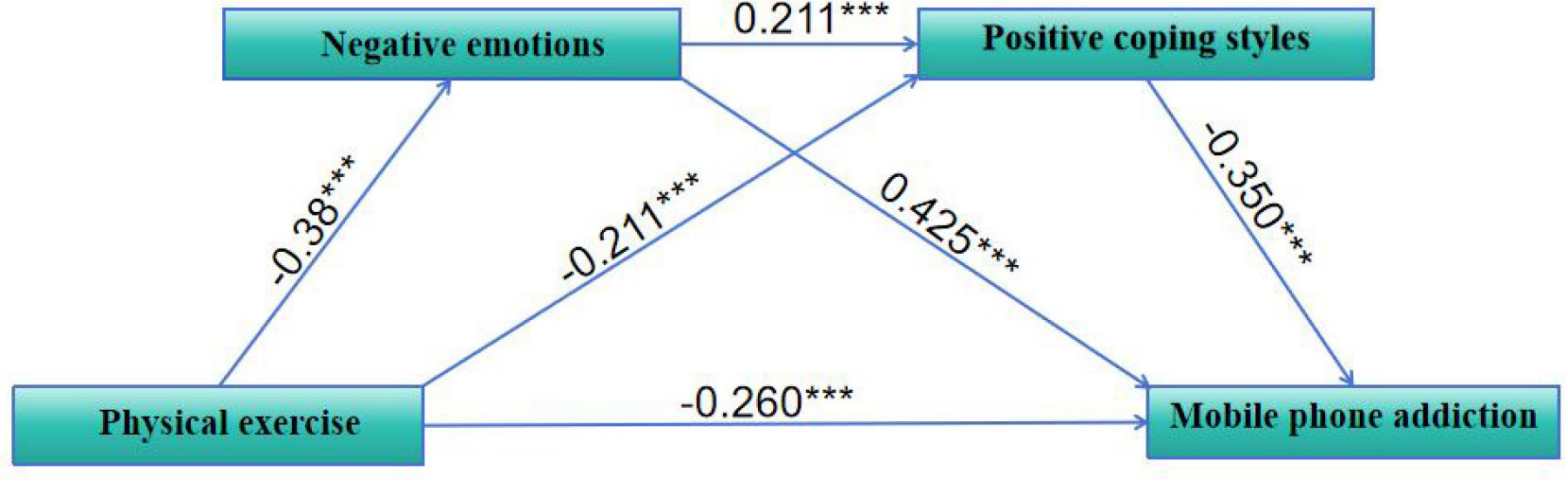
The chain mediation model of negative emotions—positive coping styles. ***p < 0.001.

This study examined how negative emotions and negative coping styles serially mediate the physical exercise and mobile phone addiction link in college students. Results (Table 4) showed a significant total effect. With mediators included, physical exercise was negatively associated with negative emotions (β = −0.380, *t* = −31.667, *p* < 0.001) and negative coping styles (β = −0.207, *t* = −34.5, *p* < 0.001). Negative emotions, in turn, positively influenced negative coping styles (β = 0.350, *t* = 38.889, *p* < 0.001) and mobile phone addiction (β = 0.437, *t* = 21.85, *p* < 0.001). Negative coping styles also positively predicted mobile phone addiction *(*β = 0.182, *t* = 5.056, *p* < 0.001). The direct effect remained significant (β = −0.254, *t* = −12.7, *p* < 0.001) after controlling for gender.

**Table 4:** Regression models for the mediating effects of negative emotions and negative coping styles.

Regression equation			Regression coefficients			*P*
Outcome variable	Predictive variable	*R* ^2^	SE	β	*t*	
Negative emotions	Physical exercise	0.144	0.012	−0.380	−31.667^***^	< 0.001
Negative coping styles	Physical exercise	0.011	0.006	−0.207	−34.500^***^	< 0.001
	Negative emotions	0.011	0.009	0.350	38.889^***^	< 0.001
Mobile phone addiction	Physical exercise	0.039	0.014	−0.254	−18.571^***^	< 0.001
	Negative emotions	0.039	0.020	0.437	21.850^***^	< 0.001
	Negative coping styles	0.039	0.036	0.182	5.056^***^	< 0.001

^***^P < 0.001.

Mediation results (Table 5; Figure 4) revealed a total effect of −0.933 (95% CI [−1.149, −0.493]), consistent with H1, higher exercise levels were statistically associated with lower mobile phone addiction among college students. The direct effect (−0.201, 95% CI [−0.451, −0.069]) accounted for 21.54% of the total. Specifically, the chain indirect effect amounted to −0.732 (95% CI [−0.833, −0.420]). The statistical significance of this mediation is confirmed by the confidence interval, which does not span zero. This indirect pathway explains 78.46% of the total effect. The marginal difference in total-indirect-effect proportions between the two models originates from variation within the negative-emotion pathway rather than from coping style specific paths. Further decomposition identified three specific indirect pathways: path 1: Physical exercise → Negative emotions → Mobile phone addiction (Effect = −0.686, 95% CI [−0.858, −0.451]); path 2: Physical exercise → Negative coping styles → Mobile phone addiction (Effect = −0.033, 95% CI [−0.065, −0.008]); path3: Physical exercise → Negative emotions → Negative coping styles → Mobile phone addiction (Effect = −0.013, 95% CI [−0.017, −0.003]). Contrast analyses between pairs of paths demonstrated significant variations in effect strength. Given that all intervals excluded zero, it is confirmed that the Physical exercise → Negative emotions → Mobile phone addiction pathway exerts a significantly stronger influence than the alternatives.

**Table 5:** Tests of mediation effects between physical exercise and mobile phone addiction among college students.

Path	Effect	Boot SE	Boot 95% CI	Proportion (%)
Total effect	−0.933	0.167	[−1.149, −0.493]	100
Direct effect	−0.201	0.133	[−0.451, −0.069]	21.54
Total indirect effect	−0.732	0.106	[−0.833, −0.420]	78.46
Path 1	−0.686	0.103	[−0.858, −0.451]	73.53
Path 2	−0.033	0.014	[−0.065, −0.008]	3.53
Path 3	−0.013	0.002	[−0.017, −0.003]	1.4

**Figure 4:**
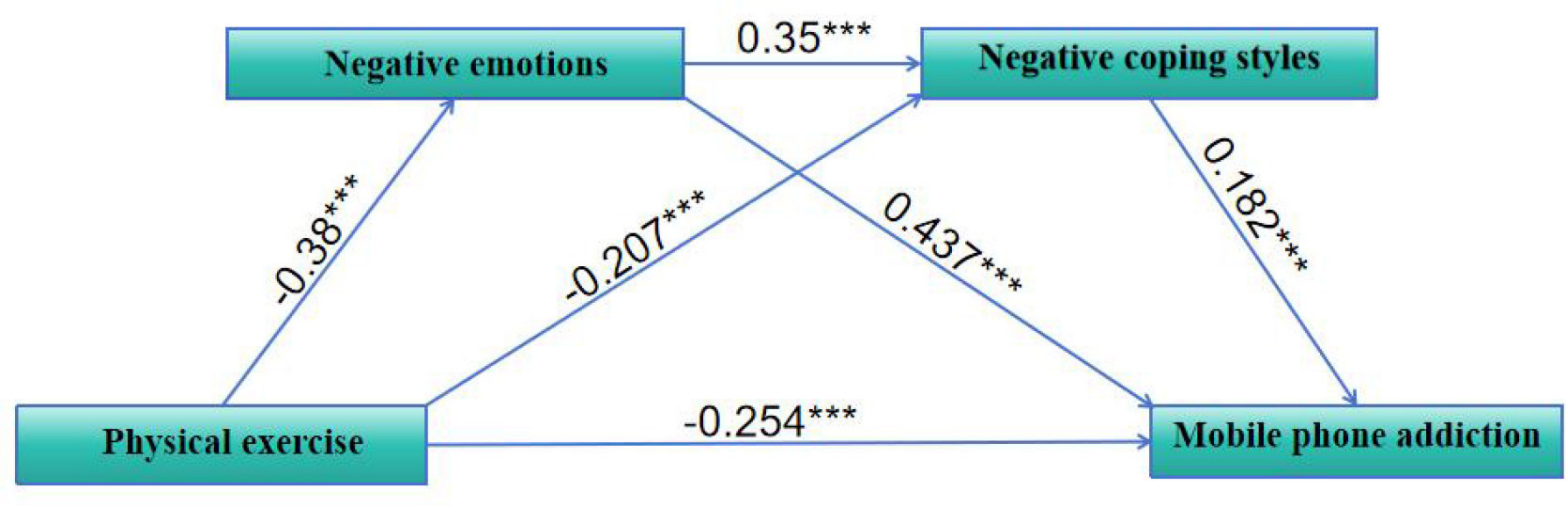
The chain mediation model of negative emotions—negative coping styles. ***p < 0.001.

## Discussion

The present investigation constructed and verified a serial chain mediation model to clarify the psychological mechanisms underlying the association between physical exercise and college students' mobile phone addiction, with correlational empirical results consistent with all proposed hypothesized associational pathways. Specifically, regular physical exercise participation was associated with lower mobile-phone addiction risk in this sample (H1), alongside two distinct indirect pathways: the independent mediating effect of negative emotions (H2), and the serial mediating path from negative emotions to coping styles (H4). Moreover, obvious discrepancies existed between positive and negative coping in their respective mediating effects. Given the large sample size of this study, we prioritized correlation magnitude (effect size) rather than statistical significance when interpreting variable relationships. Detailed analyses of these core findings are presented below.

### Direct effect of physical exercise events on mobile phone addiction

Drawing upon cross-sectional data, this study preliminarily indicates that physical exercise is inversely related to mobile phone addiction among undergraduates, a finding that aligns with established research, Thus, Hypothesis H1 was fully supported. College students are in a key developmental period and experience various stressors. With less external supervision than before, they are more likely to exhibit self-control difficulties, which may gradually result in psychological problems (Li et al., 2024a; Tian et al., 2025). In correlational patterns, routine physical exercise participation is linked to better physical health, higher positive affect and lower occurrence of unhealthy behaviors including sedentary lifestyles and excessive mobile phone use. Physical exercise demands continuous concentration on technical performance, rule compliance, and collaborative coordination (Peng et al., 2025). In correlational patterns, higher physical exercise levels occur with lower mobile phone use time and desire. Theoretically, long-term regular physical exercise may reshape brain neural pathways via repeated attention switching and behavioral reinforcement, which is a hypothesized mechanism for developing stable patterns of reduced mobile phone use; nevertheless, such causal interpretations cannot be drawn from our cross sectional data. This is consistent with the assertion of relevant studies that “physical exercise can break the vicious cycle of loneliness-mobile phone addiction-sleep disorders” (Bo, 2022). Cross-sectional data further corroborate this inverse relationship: mobile phone addiction scores were significantly lower in the sufficient vs. insufficient exercise group. Logistic regression identified addiction severity as a statistical predictor of insufficient exercise (OR = 1.022, 95% CI [1.008, 1.036]) (Liu et al., 2025). This finding further suggests a potential negative correlational pattern between mobile phone addiction severity and physical exercise engagement among college students. Given the sample characteristics, the overall physical exercise participation rate among college students remains generally low (with an average score of 18.49 on the Physical exercise Level Scale, indicating low activity levels). The marked individual differences in mobile phone addiction further underscore the practical significance of the direct intervention effect of physical exercise. Furthermore, these correlational patterns may provide preliminary reference for universities designing prevention oriented programs for mobile phone addiction. By encouraging students to enhance exercise frequency and intensity without relying on complicated psychological therapies, initial prevention can be accomplished, providing a basis for constructing a multi-level intervention framework.

From the perspective of Self-Determination Theory, physical exercise is an effective way to satisfy individuals' three core innate psychological needs: autonomy, relatedness and competence. During group or individual physical activities, college students gain a sense of autonomy by freely arranging exercise content and rhythm, build interpersonal relatedness through interaction with peers, and acquire a strong sense of competence by achieving exercise goals and improving physical fitness. When these basic psychological needs are fully met in real-life physical activities, college students no longer rely on mobile phones as a compensatory tool to make up for unmet psychological needs, which fundamentally weakens their dependence on mobile phones and explains the direct negative association between physical exercise and mobile phone addiction observed in this study.

### Mediating effect of negative emotions

In our correlational data, physical-exercise levels were linked to mobile-phone addiction via multiple pathways, among which negative emotions constituted the primary mediating variable. Path effect comparisons confirmed that negative emotion's independent mediation exceeds both coping styles and the serial chain, clarifying its central role in the physical exercise and mobile phone addiction link. Specifically, lower levels of physical exercise are associated with higher negative-emotion levels, which in turn correlate with greater addiction risk—supporting H2. Grounded in Emotion Regulation Theory, the hypothesized buffering association between exercise and negative emotions may operate through well-defined biopsychological pathways (Li et al., 2023). On the neurochemical front, regular physical exertion upregulates the availability of catecholamines such as dopamine and norepinephrine, while simultaneously promoting the release of endorphins. These neurochemicals target central nervous system regions, modulating emotional circuitry to inhibit negative emotions generation and accumulation (Gao et al., 2012). However, according to neurobiological perspectives, insufficient physical activity may constrain neurotransmitter availability, which could contribute to vulnerability to persistent negative emotions when students confront stressors such as academic and interpersonal stress (Zhu et al., 2024). At the psychological level, Social Conflict Theory posits that physical exercise can be endowed with the functions of a “stress reducer” and a “safety valve,” which can help individuals release aggressive instincts, alleviate emotional blockages, and further reduce the accumulation of negative emotions (Xie et al., 2023). In contrast, college students who lack physical exercise are deprived of this effective emotional catharsis channel. From the perspective of this hypothesized associational pathway, negative emotional experiences under stress are associated with greater tendencies to seek virtual comfort via mobile phones among college students (Chen et al., 2025; Yuan et al., 2024). The correlation analysis in this study showed that the correlation coefficient between negative emotions and mobile phone addiction was as high as 0.618, indicating a strong association between the two. This is consistent with the conclusions of relevant cross-sectional studies, which found that individuals with severe internet addiction (mainly manifested in mobile phone use) had significantly higher scores on negative emotions such as anxiety and depression than non-addicted individuals (Chen and Zhang, 2024). For college students whose self-control ability is not yet fully developed, such behavior of escaping real negative emotions through mobile phones can easily transform from occasional emotional compensation into long-term addictive dependence, ultimately exacerbating the tendency of mobile phone addiction. In addition, a comparison of the results of the two mediation models revealed that regardless of the type of subsequent coping styles, the proportion of the mediating effect of negative emotions remained stable at around 80%. This characteristic indicates that negative emotions are the core transmission node of the impact of physical exercise on mobile phone addiction, and their role priority is higher than that of coping styles, which also provides new empirical evidence for relevant studies emphasizing the importance of emotional regulation in addiction intervention. From a practical perspective, the mediating mechanism of negative emotions provides a precise entry point for universities to develop mobile phone addiction intervention programs. Not only should efforts focus on alleviating negative emotions at the source by increasing physical exercise participation, but also on offering emotional regulation courses to help college students master scientific methods of emotional guidance. This approach reduces their tendency to use mobile phones as an escape from emotional distress, which may theoretically interrupt the hypothesized correlational chain. The statistical comparison of pathway effects further quantitatively confirms the dominant role of this pathway, providing robust guidance for the formulation of subsequent intervention plans.

Combined with Self-Determination Theory, unmet basic psychological needs are an important source of negative emotions among college students. When students lack effective physical activity and cannot obtain autonomy, relatedness and competence from real life, they will continuously generate anxiety, depression and other negative emotions. To relieve such unpleasant emotional experiences, they turn to mobile network platforms to pursue instant psychological satisfaction and compensate for psychological deficits, which gradually evolves into mobile phone addiction. These correlational patterns illustrate why negative emotions occupy a central position within the observed associational network: mismatches between physical-activity-related psychological-need satisfaction and psychological states co-occur with emotional distress, which further correlates with problematic mobile-phone-use behaviors.

### Mediating effect of coping styles

The correlational data preliminarily indicate coping styles may act as one potential intermediate linking physical exercise and mobile phone addiction. Notably, heterogeneous patterns were observed when comparing the mediating mechanisms of positive vs. negative coping strategies, indicating distinct functional characteristics for each. Hypotheses H3, H3a, and H3b were fully supported. The path effect difference test also found that the independent and chain mediation path effects of coping styles were significantly lower than that of negative emotion. This result indicates that coping styles is an important transmission node of physical exercise affecting mobile phone addiction, but its role is secondary, and it needs to rely on the regulation of negative emotion to play a better role. Examination of the data indicates that physical exercise levels are associated with greater adoption of constructive coping styles and lower tendency toward destructive ones. Consequently, individuals employing adaptive strategies exhibit diminished mobile phone dependency, whereas those relying on maladaptive tactics face heightened vulnerability. This empirical evidence reinforces the established theoretical framework regarding coping styles as determinants of problematic mobile use (Zu et al., 2016). From the analysis of specific action paths and mechanisms, physical exercise can systematically enhance individuals' stress regulation ability and psychological resilience (Blevins et al., 2017; Thome and Espelage, 2004), prompting them to prefer positive coping strategies such as problem-solving and active social support-seeking when facing various stressful events like academic stress and interpersonal conflicts. College students themselves tend to favor positive coping patterns in stress management, and physical exercise can transfer the frustration-resistant experience and collaborative ability gained during sports to real-life scenarios, further consolidating their inclination toward positive coping. As a result, the psychological compulsion to retreat from reality into the virtual world is lessened, which in turn decreases the probability of falling into mobile phone addiction (Cheng et al., 2023). Nevertheless, the independent indirect effect of positive coping was small. In line with prior work, higher negative-emotion scores correlated with lower endorsement of positive coping strategies, which in turn showed weaker correlational links with mobile-phone-addiction indicators (He et al., 2020).

Regarding maladaptive coping mechanisms, physical exercise engagement correlates with lower reliance on avoidant coping strategies such as avoidance and self-blame. This beneficial effect is potentially driven by the improvement of individuals' self-efficacy and their capacity for emotion regulation, thereby fostering more adaptive psychological responses (Chen et al., 2024; Scully et al., 1998), but the intensity of this inhibitory effect is relatively weak. Meanwhile, the accumulation of negative emotions significantly strengthens individuals' tendency toward negative coping, and the two factors jointly influence college students' choice of coping strategies (Tang et al., 2013). The independent mediating proportion associated with positive coping styles was slightly larger than that of negative coping styles. Nevertheless, the effect proportions for both coping-style-related independent pathways were small compared with the dominant negative-emotion pathway. Additionally, the association between negative coping and addiction is prone to disruption by other psychological factors. Nevertheless, negative coping styles remain a critical pathway through which physical exercise influences mobile phone addiction. Specifically, within the mediating system formed alongside negative emotions, they can fully transmit the indirect effects of exercise on mobile phone addiction (Cheng et al., 2023). Overall, although the mediating effect of coping styles was weaker than that of negative emotions and had certain regulatory complexity, it was still an indispensable transmission node between physical exercise and mobile phone addiction. Its differentiated effect patterns also reflected the inherent complexity of the association between college students' coping strategy choices and mobile phone addiction, providing a precise entry point for subsequent stratified and classified addiction interventions (Thome and Espelage, 2004). The statistical comparison of path effects more clearly quantifies the strength of the mediating role of coping styles, suggesting that universities may adopt the cultivation of coping styles as an auxiliary pathway when implementing interventions.

From the perspective of Self-Determination Theory, the satisfaction of basic psychological needs reshapes individuals' tendency to adopt different coping styles. When college students gain sufficient autonomy, relatedness and competence through physical exercise, their internal psychological resources are enriched, which helps them actively face difficulties and choose positive coping strategies rather than escaping from pressure. On the contrary, long-term unmet psychological needs will make individuals trapped in passive states, and they are more likely to adopt avoidance and other negative coping methods. Mobile phones then become a convenient escape carrier for negative coping behaviors, which explains why coping styles play a secondary mediating role between physical exercise and mobile phone addiction.

### Chain-mediated effects of physical exercise and coping styles

The path effect difference test revealed that the chain-mediated pathway's effect strength was not only significantly lower than the independent mediation pathway of negative emotions, but also marginally weaker than that of coping styles. This indicates that while the chain transmission between negative emotions and coping styles serves as an effective pathway for physical exercise to influence mobile phone addiction, its synergistic effect remains limited, with the core mechanism still primarily being the independent mediation of negative emotions. Furthermore, a significant bidirectional interaction exists between negative emotions and coping styles. When confronted with academic, interpersonal, or other stressors, individuals are more likely to abandon adaptive coping strategies—such as problem-solving and seeking social support—and instead resort to maladaptive approaches like self-blame and avoidance (Deng et al., 2023; Geng et al., 2008), thereby reducing their willingness to engage in active coping (Deng et al., 2023). On the other hand, negative coping styles further exacerbate the accumulation of negative emotions through the path of “escaping reality—accumulating problems,” forming a vicious cycle of “negative emotions → negative coping → more severe negative emotions” (Xie et al., 2016). For college students with insufficient physical exercise, who are inherently more prone to negative emotional accumulation, falling into the aforementioned vicious cycle will drive them to seek virtual comfort through mobile phones to escape real stress, ultimately resulting in a significant aggravation of mobile phone addiction (Tu and Guo, 2011). Correlation analysis reveals correlational patterns suggesting physical exercise is associated with mobile phone addiction following a sequential chain pathway: by first reducing negative emotional states and subsequently optimizing the selection of coping styles. Specifically, physical exercise levels correlate with lower negative emotion levels and greater positive coping tendency, which may theoretically interrupt the hypothesized pathway from negative emotions to mobile phone addiction. The obtained correlational results are consistent with the theoretical expectation of Hypothesis H4. Although this serial chain is statistically detectable, its effect magnitude is very small. The co-occurrence of negative emotions and coping styles reflects a plausible theoretical synergistic mechanism. Combined with the core viewpoints of Self-Determination Theory, the complete chain mechanism of physical exercise → negative emotions → coping styles → mobile phone addiction essentially reflects the whole process of psychological need deprivation evolving into problematic behaviors. From a theoretical standpoint rooted in Self-Determination Theory, insufficient satisfaction of autonomy, relatedness and competence needs may trigger persistent negative emotions. Driven by negative emotions, individuals may prefer negative coping strategies to evade real world pressure; mobile phones as low-threshold compensatory outlets may contribute to addictive like behaviors.

## Conclusion

Based on cross-sectional correlational data, this study preliminarily suggests physical exercise may have a potential negative correlation with college students' mobile phone addiction. Negative emotions and two subtypes of coping styles act as intermediate variables in such correlational relations, and the indirect path centered on negative emotions accounts for the largest effect proportion in this sample. Path comparison shows the chain-model explanatory proportions for both coping subtypes remain very low. The dominant explanatory power originates from the negative-emotion independent mediation path. These correlational results provide quantitative reference for designing sports-related prevention strategies for high-risk students.

## Limitations of the study

This study has several limitations. First, the cross sectional design precludes causal inferences. Although serial mediating associations were observed, bidirectional relationships among variables cannot be ruled out, so we can only interpret the findings as correlational. Second, convenience sampling and self-report measures may introduce selection and social desirability biases. Furthermore, common method bias was only evaluated via Harman's single factor test; the more rigorous latent method factor CFA was not performed. Future research should employ longitudinal or experimental designs, multi-source data and objective indicators to clarify causal directions and reduce method-related biases.

## Data Availability

The raw data supporting the conclusions of this article will be made available by the authors, without undue reservation.
